# Personalised learning in higher education for health sciences: a scoping review protocol

**DOI:** 10.1186/s13643-024-02478-4

**Published:** 2024-04-02

**Authors:** Majid Ali, Izyan Binti A. Wahab, Hasniza Zaman Huri, Muhamad Saiful Yusoff

**Affiliations:** 1 Department of Basic Sciences, College of Medicine, Sulaiman Al-Rajhi University, Al-Bukayriyah, Saudi Arabia; 2 Department of Clinical Pharmacy and Pharmacy Practice, Faculty of Pharmacy, Universiti Malaya, Kuala Lumpur, Malaysia; 3 Department of Medical Education, School of Medical Sciences, Universiti Sains Malaysia, Kota Bharu, Kelantan, Malaysia

**Keywords:** Personalised learning, Customised learning, Individualised learning, Adaptive learning, Tailored learning, Learner-centred learning, Student-centred learning, Individualised instructions, Learning preferences, University, Health sciences, Higher education, Scoping review

## Abstract

**Background:**

Personalised learning, an educational approach that tailors teaching and learning to individual needs and preferences, has gained attention in recent years, particularly in higher education. Advances in educational technology have facilitated the implementation of personalised learning in various contexts. Despite its potential benefits, the literature on personalised learning in health sciences higher education remains scattered and heterogeneous. This scoping review aims to identify and map the current literature on personalised learning in health sciences higher education and its definition, implementation strategies, benefits, and limitations.

**Methods:**

A comprehensive search of electronic databases, PubMed, Scopus, Google Scholar, Educational Research Complete, and Journal Storage (JSTOR), will be conducted to identify relevant articles. The search will be limited to articles published in the English language between 2000 and 2023. The search strategy will be designed and adapted for each database using a combination of keywords and subject headings related to personalised learning and health sciences higher education. Eligibility criteria will be applied to screen and select articles. Data extraction and quality assessment will be performed, and thematic synthesis will be used to analyse the extracted data.

**Discussion:**

The results of the scoping review will present a comprehensive and coherent overview of the literature on personalised learning in health sciences higher education. Key themes and topics related to personalised learning, its definitions, models, implementation strategies, benefits, and limitations, will be identified. The geographical and temporal distribution of research on personalised learning in health sciences higher education will also be described. This scoping review will provide a structured synthesis of the available evidence on personalised learning in health sciences higher education, highlighting potential gaps and areas for future research. The findings will contribute to ongoing scholarly and policy debates on personalised learning in higher education, informing the development of best practices, guidelines, and future research agendas.

## Introduction

### Background

Personalised learning is an educational approach that tailors the teaching and learning process to the individual needs and preferences of learners [1–3]. This approach is rooted in the constructivist theories of education, emphasising the importance of active, self-directed learning, and the development of personalised learning pathways. It has gained considerable attention in recent years as educators and researchers recognise the importance of addressing the diverse needs of students in order to improve learning outcomes [4]. In higher education, the traditional one-size-fits-all teaching methods may not be effective for all students, as they often fail to accommodate individual differences in learning styles, abilities, and interests [5]. Advances in educational technology, such as learning management systems, adaptive learning platforms, and learning analytics, have further facilitated the implementation of personalised learning in various educational contexts [6, 7].

The concept of personalised learning can be traced back to the early twentieth century when educational theorists like John Dewey emphasised the importance of individualised instruction [8]. Since then, numerous definitions and models of personalised learning have emerged, reflecting the diverse perspectives and approaches to implementing this concept in educational settings [1]. In general, personalised learning can be characterised by the customization of learning activities, materials, and assessments to meet the unique needs and preferences of individual learners [9]. This may involve the use of digital technologies, such as adaptive learning platforms, to provide real-time feedback and tailor instructional content based on student performance and progress [10]. Some studies leveraged learning analytics to gain meaningful insights into learning processes and student development [11, 12]. In their work, Gašević and colleagues tackled the difficulties and concerns linked to the real-world implementation of learning analytics in educational research, aiming to capture its lasting effects on student learning and teaching methods [11]. They emphasised the potential of analytics to provide personalised feedback, facilitate decision-making, and enhance overall learning outcomes. Siemens delved into the theoretical foundations, methodologies, and practical applications of learning analytics [12]. He emphasised the significance of analytics in analysing large volumes of educational data and extracting valuable insights to inform decision-making, instructional design, and personalised learning approaches.

The potential benefits of personalised learning in higher education have been widely acknowledged in the literature. For instance, personalised learning has been associated with improved student engagement, satisfaction, and retention rates, as well as increased academic performance and the development of critical thinking and problem-solving skills [13, 14]. Moreover, personalised learning can help address the needs of diverse student populations, including those with learning disabilities, English language learners, and nontraditional students [15, 16]. Alamri and colleagues conducted a study which explored the implementation and effectiveness of personalised learning environments in higher education [17]. The authors emphasised the potential of personalised learning to tailor the instructions according to individual learners’ preferences, needs, and learning styles. The study highlighted positive outcomes such as increased student engagement, motivation, and improved learning outcomes resulting from the adoption of personalised learning. Several studies have reported positive outcomes associated with personalised learning interventions, such as improved self-regulation, metacognitive skills, and learning outcomes [3, 4, 15, 18, 19]. Despite these potential benefits, there are also challenges and limitations associated with personalised learning, such as the cost and complexity of implementing adaptive learning technologies, the potential for an increased digital divide, and concerns regarding student privacy and data security [6, 7]. Moreover, the literature on personalised learning in health sciences higher education remains scattered and heterogeneous, with various definitions, models, and methods being proposed and implemented across different fields and disciplines [1].

Given the potential of personalised learning to address the challenges and opportunities of contemporary higher education, there is a need for a comprehensive and structured synthesis of the available evidence on this topic. Despite the competitive courses offered in health sciences, the knowledge of personalised learning in higher education remains scarce and limited. The in-depth knowledge and understanding of personalised learning can enhance the learning strategy methods and hence alleviate the challenging nature of health sciences courses and thus diminish students’ attrition rate. Scoping reviews are a suitable methodology for this purpose, as they aim to identify and map the key concepts, theories, and sources of evidence in a given research area, providing a broad overview of the literature and identifying research gaps [20, 21]. Therefore, this scoping review aims to identify the current literature on personalised learning in higher education for health sciences (medicine, pharmacy, nursing, dentistry, physiotherapy, and radiology), including its definition, implementation, benefits, and limitations.

#### Objectives

The primary objectives of this scoping review are as follows:

- Identify definitions of personalised learning in the health sciences higher education context.
- Examine the implemented strategies of personalised learning and their evaluation (including topics/fields related to personalised learning) in the health sciences higher education context.
- Outline the benefits and limitations of personalised learning in the health sciences higher education context.
- Discuss the implications of personalised learning in the health sciences higher education context.

## Methods

### Study design

This scoping review protocol will follow the methodological framework outlined by Arksey and O'Malley [20] and the guidance provided in the JBI Manual for Evidence Synthesis [22]. The review will be reported according to the PRISMA Extension for Scoping Reviews (PRISMA-ScR) guidelines [23].

### Information sources and search strategy

A comprehensive search of electronic databases will be conducted to identify relevant literature. The following databases will be searched: PubMed, Scopus, Google Scholar, Educational Research Complete, and Journal Storage (JSTOR). The search strategy will be designed and adapted for each database using a combination of keywords and subject headings related to personalised learning and health sciences higher education. The following keywords will be utilised in various combinations, employing BOOLEAN operators to refine the search: “Personalised learning”, “Individualised learning”, “Customised learning”, “Tailored learning”, “Adaptive learning”, “Individualised instructions/guide”, “Personalised instructions/guide”, “Learning preferences”, “Student-centred learning/instructions”, “Learner-centred learning/instructions”, “Health sciences”, “Healthcare sciences”, “Higher education”, “College”, “University”, and “Academia”.

An example search string for PubMed will be as follows: (("personalised learning" OR "individualised learning" OR "customised learning" OR "tailored learning" OR "adaptive learning" OR "individualised instructions" OR "individualised guide" OR "personalised instructions" OR "personalised guide" OR "learning preferences" OR "student-centred learning" OR "student-centred instructions" OR "learner-centred learning" OR "learner-centred instructions" OR "Self-directed learning as topic/classification"[MeSH] OR "Self-directed learning as topic/ethics"[MeSH]) AND ("health sciences" OR "healthcare sciences" OR "health care category"[MeSH]) AND ("higher education" OR "college" OR "university" OR "academia" OR "education, graduate"[MeSH])).

All searches will be limited to articles published in English between 2000 and 2023. The reference lists of included literature/articles will also be hand-searched to identify additional relevant studies.

### Eligibility criteria

Inclusion criteria are as follows:

- Published in the English language
- Study design: All study types
- Study location: From all geographical locations
- Focused on personalised learning in higher education [health sciences (medicine, pharmacy, nursing, dentistry, physiotherapy, radiology)]
- Published between 2000 and 2023
- Both peer-reviewed and non-peer-reviewed

Exclusion criteria were as follows:

- Published in a language other than English language
- Not focused on personalised learning in health sciences higher education
- Published before 2000

### Selection process

The screening and selection process will involve several steps to ensure the comprehensive and systematic identification of relevant literature. First, the search results from each database will be imported into reference management software (e.g. EndNote) to remove duplicates. Two reviewers will screen the titles and abstracts of the identified articles against the eligibility criteria independently. Full-text assessments will be performed for potentially eligible articles, and any disagreements between the reviewers will be resolved through discussion or consultation with a third reviewer. The reasons for excluding articles during the full-text screening will be recorded, and a PRISMA flow diagram will be used to illustrate the selection process [24].

### Data extraction

A standardised data extraction form will be developed based on JBI guidelines and pilot-tested on a subset of included articles [22]. Any modifications in the data extraction form following the piloting will be reported. The following data will be extracted from each article:

- Authors and year
- Country/geographical area
- Title
- Aim
- Healthcare field (medicine, nursing, pharmacy, dentistry, physiotherapy, radiology)
- Study population/sample size
- Topic (learning and teaching, assessment, feedback)
- Study design/tools/intervention
- Key findings
- Research gap

Data extraction will be performed by the same two reviewers independently, with any discrepancies resolved through discussion or consultation with a third reviewer. The reviewers may contact the authors of selected papers for any clarifications.

### Quality assessment

Although not a mandatory step in scoping reviews, the quality assessment of the included articles will be conducted using the tool developed by Kmet and colleagues [25]. This tool provides a set of standardised criteria to assess the methodological quality of primary research papers from various fields. The quality assessment will help identify potential sources of bias, assess the rigour of the study designs, and provide insights into the overall quality of the available evidence on personalised learning in health sciences higher education. The quality assessment will be conducted by the same two reviewers independently, with any discrepancies resolved through discussion or consultation with a third reviewer.

### Data synthesis

A thematic synthesis approach will be used to present and analyse the extracted data, following the steps outlined by Thomas and Harden [26]. This will involve the following stages: (1) familiarisation with the data, (2) development of descriptive themes, (3) generation of analytical themes, and (4) refinement and synthesis of themes. The key themes and topics related to personalised learning in health sciences higher education, including definitions, models, and implementation strategies, will be identified. Additionally, the benefits and limitations of personalised learning in health sciences higher education will be summarised. The results reported will be discussed and approved by all the reviewers.

### Ethical considerations

As this scoping review will include publicly available published material, ethical approval is not required.

## Results

The results of the scoping review will be presented in a narrative format, following the PRISMA-ScR guidelines [22], providing a comprehensive and coherent overview of the literature on personalised learning in health sciences higher education. This will include the definitions, key themes and topics, and implementation strategies with benefits and limitations of personalised learning in health sciences higher education, along with a critical appraisal of the methodological quality and rigour of the included articles. The geographical and temporal distribution of the research on personalised learning in health sciences higher education will also be described, highlighting potential gaps and areas for future research. The results are expected to be presented in a full scoping review in 2024.

## Discussion

The “Discussion” section will delve into the key findings of the scoping review, exploring the implications of these findings for the broader field of health sciences in higher education research and practice. In particular, we will examine the various definitions and models of personalised learning that have been proposed in the literature, discussing the extent to which they align with or diverge from one another. We will also consider the potential reasons for these differences, such as the influence of specific educational contexts, technologies, or pedagogical approaches.

Furthermore, we will discuss the benefits and limitations of personalised learning in health sciences higher education as reported in the literature. We will analyse the factors that may contribute to the success or failure of personalised learning initiatives, such as the role of institutional support, faculty engagement, and student motivation. We will also explore the potential risks and challenges associated with personalised learning, such as concerns related to privacy, equity, and the digital divide.

The discussion will also address the methodological issues and limitations of the existing research on personalised learning in health sciences higher education. We will critically appraise the quality of the included articles using the tool developed by Kmet and colleagues [25], considering the potential sources of bias, the rigor of the study designs, and the overall quality of the available evidence. We will identify any gaps in the literature and areas where further research is needed to enhance our understanding of personalised learning in health sciences higher education.

### Expected outcomes

Through this scoping review, we aim to achieve the following outcomes:

- Provide a comprehensive and structured overview of the literature on personalised learning in higher education for health sciences, including its definition, implementation, benefits, and limitations.
- Identify the key themes and topics related to personalised learning in health sciences higher education, offering a clear and coherent synthesis of the available evidence.
- Highlight the geographical and temporal distribution of research on personalised learning in health sciences higher education, identifying potential gaps and areas for future research.
- Highlight the differences or comparisons between different topics/fields related to personalised learning in health sciences higher education.
- Offer a critical appraisal of the methodological quality and rigour of the included articles, providing insights into the overall quality of the available evidence on personalised learning in health sciences higher education.
- Contribute to the ongoing scholarly and policy debates on personalised learning in higher education for health sciences, informing the development of best practices, guidelines, and future research agendas.

## Conclusion

By achieving these outcomes, we hope to advance the field of personalised learning in higher education for health sciences, providing a solid foundation for future research and practice. We anticipate that the results of this scoping review will be of interest to a wide range of stakeholders, including researchers, educators, administrators, policymakers, and students, who are keen to understand and harness the potential of personalised learning to enhance teaching and learning in higher education.

## Data Availability

The datasets and/or any supplementary material used in this scoping review will be available from the corresponding author upon request.
